# Constraints of Weight Loss as a Marker of Bariatric Surgery Success: An Exploratory Study

**DOI:** 10.3389/fphys.2021.640191

**Published:** 2021-06-11

**Authors:** Saulo Gil, Karla Goessler, Wagner S. Dantas, Igor Hisashi Murai, Carlos Alberto Abujabra Merege-Filho, Rosa Maria R. Pereira, Roberto de Cleva, Marco Aurélio Santo, John P. Kirwan, Hamilton Roschel, Bruno Gualano

**Affiliations:** ^1^Applied Physiology and Nutrition Research Group, School of Physical Education and Sport, Faculdade de Medicina FMUSP, Universidade de São Paulo, São Paulo, Brazil; ^2^Integrated Physiology and Molecular Medicine Laboratory, Pennington Biomedical Research Center, Baton Rouge, LA, United States; ^3^Rheumatology Division, Hospital das Clinicas HCFMUSP, Faculdade de Medicina, Universidade de São Paulo, São Paulo, Brazil; ^4^Department of Digestive Surgery, Hospital das Clinicas HCFMUSP, Faculdade de Medicina, Universidade de São Paulo, São Paulo, Brazil

**Keywords:** gastric bypass, cardiometabolic risk (factors), metabolic health, obesity, weight loss

## Abstract

**Purpose:**

The aim of this exploratory study was to investigate whether the degree of weight loss properly reflects improvements in cardiometabolic health among patients who underwent Roux-en-Y gastric bypass.

**Methods:**

In this ancillary analysis from a clinical trial, patients were clustered into tertiles according to the magnitude of the percentage weight loss (1st tertile: “higher weight loss”: −37.1 ± 5.8%; 2nd tertile: “moderate weight loss”: −29.7 ± 1.4%; 3rd tertile: “lower weight loss”: −24.2 ± 2.3%). Delta changes (9 months after surgery-baseline) in clustered cardiometabolic risk (i.e., blood pressure index, fasting glucose, high-density lipoprotein [HDL] and triglycerides [TG]), glycated hemoglobin (HbA1c), homeostasis model assessment (HOMA-IR), and C-reactive protein (CRP) were calculated.

**Results:**

A total of 42 patients who had complete bodyweight data (age = 40 ± 8 year; BMI = 47.8 ± 7.1 kg/m^2^) were included. Surgery led to substantial weight loss (−37.9 ± 11.3 kg, *P* < 0,001), and clinically significant improvements in blood pressure index (−17.7 ± 8.2 mmHg, *P* < 0.001), fasting glucose (−36.6 ± 52.5 mg/dL, *P* < 0.001), HDL (9.4 ± 7.1 mg/dL, *P* < 0.001), TG (−35.8 ± 44.1 mg/dL *P* < 0,001), HbA1c (−1.2 ± 1.6%, *P* < 0.001), HOMA-IR (−4.7 ± 3.9 mg/dL, *P* < 0.001) and CRP (−8.5 ± 6.7 μg/mL *P* < 0.001). Comparisons across tertiles revealed no differences for cardiometabolic risk score, fasting glucose, HbAc1, HOMA-IR, blood pressure index, CRP, HDL, and TG (*P* > 0.05 for all). Individual variable analysis confirmed cardiometabolic improvements across the spectrum on weight-loss. There were no associations between weight loss and any dependent variable.

**Conclusion:**

Weight loss following bariatric surgery does not correlate with improvements in cardiovascular risk factors. These findings suggest that weight loss alone may be insufficient to assess the cardiometabolic success of bariatric surgery, and the search for alternate proxies that better predict surgery success are needed.

## Introduction

Bariatric surgery is the preferred treatment for morbid obesity because it yields substantial and sustained weight loss, and reduces the severity of cardiometabolic risk factors (Schauer et al., 2017; Schiavon et al., 2017). The magnitude of weight loss after bariatric surgery is considered indicative of treatment success (Maggard et al., 2005). However, there is still a large number (∼15–35%) of patients undergoing bariatric surgery who fail to meet clinical meaningful weight loss goals, or even experience weight regain when evaluated 12 months after surgical intervention (Magro et al., 2008; Colquitt et al., 2014). These variations may be explained by multiple factors, but changes in behavior after surgery appears to be an important determinant of success (Odom et al., 2010; Robinson et al., 2014).

Recent evidence hints that individuals with obesity who engage in lifestyle interventions (e.g., diet and exercise training) experience improvements in cardiometabolic risk factors regardless of weight loss (Ross et al., 2000; Hyde et al., 2019), suggesting that more attention should be paid to broader health markers other than weight alone in the management of obesity. Therefore, one could speculate that bariatric surgery-induced weight loss *per se* may be a marker for treatment success. This hypothesis was tested by examining whether the magnitude of weight loss induced by bariatric surgery correlates with reduced cardiometabolic risk factors.

## Materials and Methods

### Study Design and Participants

Data reported herein are derived from a large randomized controlled trial that investigated the effects of exercise training on overall cardiometabolic risk factors in individuals with obesity who had undergone bariatric surgery (clinicaltrials.gov: NCT02441361), conducted between March 2015 and June 2018. The study was approved by the local ethical committee and all patients provided written informed consent.

Inclusion criteria were as follows: women eligible for bariatric surgery (BMI > 40 kg/m^2^ or ≥ 35 kg/m^2^ with associated co-morbidities), 18–60 years of age, and not engaged in any exercise training program for at least 1 year prior to the study. Exclusion criteria involved cancer in the past 5 years, and any cardiovascular, neurological, or musculoskeletal disorders that would contraindicate exercise practice. Before surgery, patients were randomly assigned (1:1) into either standard of care, or standard of care plus exercise. All the patients underwent Roux-en-Y Gastric bypass. The exercised group performed a 6-month, supervised, exercise training program which started 3 months after surgery. Clinical and laboratory assessments were performed before (PRE), and at 3 (POST3) and 9 (POST9) months after surgery. Details regarding the experimental design, intervention, measures and outcomes, and main findings can be found elsewhere (Dantas et al., 2018; Murai et al., 2019).

In this ancillary analysis, possible differences between exercise and non-exercised groups were tested by independent *t*-test for all dependent variables [blood pressure index, fasting glucose, high-density lipoprotein (HDL) and triglycerides (TG), glycated hemoglobin (HbA_1__c_), and C-reactive protein (CRP)], and no significant differences were detected (*P* > 0.05 for all comparison; Supplementary Table 1). Thereafter, to increase the power of the analysis, exercised and non-exercised patients were examined together. Patients were clustered into tertiles according to the magnitude of the percentage weight loss (1st tertile: “higher weight loss”: −37.1 ± 5.8%; 2nd tertile: “moderate weight loss”: −29.7 ± 1.4%; 3rd tertile: “lower weight loss”: −24.2 ± 2.3%). Delta changes (POST9-PRE) in clustered cardiometabolic risk (i.e., mean arterial pressure, fasting glucose, HDL and TG, HbA_1__c_ and CRP) were calculated.

### Cardiovascular Risk Factors and Clustered Cardiometabolic Risk

Body weight was assessed using a calibrated digital scale. Cardiovascular risk factors clustered cardiometabolic risk (i.e., blood pressure index, fasting glucose, HDL and TG, HbA_1__c_ and CRP. A blood pressure index was computed by averaging systolic and diastolic pressure (Wijndaele et al., 2006). Fasting glucose was assessed using a colorimetric enzymatic assay (BioClin, Brazil). HDL, TG, and CRP were assessed using enzymatic colorimetric assays (CELM, São Paulo, Brazil). HbA_1__c_ was measured on whole blood by high-performance liquid chromatography and ion exchange using the Biorad Variant II automated analyzer (Bio-Rad^®^). Homeostasis model assessment (HOMA-IR) was calculated using the following equation: (fasting glucose in mmol × fasting insulin in μU/mL)/22.5.

An adapted, continuous, clustered cardiometabolic risk was computed using blood pressure index, fasting triglycerides, HDL, and fasting glucose (reference values were 115 mmHg, 150, 50, and 100 mg/dL, respectively) (Wijndaele et al., 2006). All variables were standardized [*z* = (value − reference)/SD)]; as for HDL (protective for cardiometabolic risk), the negative *z*-score values were converted to positive values. The risk score was the sum of all standardized scores, with higher values indicating higher cardiometabolic risk.

### Statistical Analysis

Normality of the data was assessed using a Shapiro-Wilk test. Baseline characteristics of the groups (“higher weight loss” vs. “moderate weight loss” vs. “lower weight loss”) were compared using one-way ANOVA. A dependent *t*-test was performed to compare exercised and non-exercised groups. As non-significant differences were observed between groups (*P* > 0.05), patients from the exercised and non-exercised groups were clustered into tertiles, according to their percentage of total weight loss (Table 1). Dependent *t*-tests were also used the compare the effects of surgery (POST9-PRE) on weight loss, blood pressure index, fasting glucose, HDL, TG, HbA_1__c_, HOMA-IR, and CRP. One-way ANOVA was performed to test the tertiles (“higher weight loss” vs. “moderate weight loss” vs. “lower weight loss”). Whenever a significant F value was detected, the Tukey *post hoc* test was used for multiple comparisons. Pearson product–moment linear correlation was used to test the associations between weight loss and cardiovascular risk factors and clustered cardiometabolic risk. A *post hoc*, sensitivity analysis was conducted considering changes in weight loss and cardiometabolic parameters from PRE to POST3, following the same procedures above described. Data are presented as mean ± SD. When between-group differences were detected, we also reported the estimated mean difference between groups and 95% CI. The significance level was set at *P* < 0.05. The analyses were performed using SAS^®^ version 9.3.

**TABLE 1:** Patients’ baseline (before bariatric surgery) characteristics according to weight loss tertiles.

	**Lower WL**	**Moderate WL**	**Higher WL**	***P*-value**
Sample per tertile/sample assigned to exercise* (*n*)	14/7	14/6	14/6	–
Age (years)	42.4 ± 7.3	41.9 ± 8.7	36.9 ± 7.4	0.1311
Weight (kg)	124.5 ± 22.0	128.1 ± 19.1	124.4 ± 16.9	0.8482
Clustered cardiometabolic risk (a.u.)	−5.7 ± 2.0	−6.5 ± 1.2	−7.1 ± 1.1	0.0585
Fasting glucose (mg/dL)	138.0 ± 73.3	125.4 ± 48.6	104.5 ± 22.0	0.5634
HbA_1c_ (%)	7.2 ± 2.6	6.1 ± 0.7	6.0 ± 0.8	0.0995
HOMA-IR	6.8 ± 4.6	7.5 ± 4.2	5.7 ± 2.8	0.5225
Systolic blood pressure	147.9 ± 16.1	148.3 ± 13.7	141.7 ± 15.2	0.4410
Diastolic blood pressure	90.4 ± 7.4	95.0 ± 8.8	90.0 ± 10.0	0.2601
C-reactive protein (mg/dL)	11.1 ± 5.2	12.3 ± 8.6	12.4 ± 6.8	0.8703
HDL (mg/dL)	47.6 ± 11.3	42.2 ± 14.2	40.9 ± 8.3	0.2677
Triglycerides (mg/dL)	127.3 ± 44.7	121.3 ± 50.6	119.3 ± 59.2	0.9139

Data are expressed as mean ± SD. n*: patients randomly assigned to exercise training in the clinical trial (Dantas et al., 2018; Murai et al., 2019); WL: weight loss; HbA_1c_: glycated hemoglobin; HOMA-IR: homeostasis model assessment; HDL: high-density lipoprotein; LDL: low-density lipoprotein; a.u.: arbitrary unit.

## Results

Forty-two patients who had complete data for body weight were included in this ancillary analysis (age = 40 ± 8 year; BMI = 47.8 ± 7.1 kg/m^2^; flow of participants in Supplementary Figure 1). Overall, surgery led to a large and significant decrease in weight (delta change: −37.9 ± 11.3 kg, *P* < 0.001), and improved blood pressure index (delta change: −17.7 ± 8.2 mmHg, *P* < 0.001), fasting glucose (delta change: −36.6 ± 52.5 mg/dL, *P* < 0.001), HDL (delta change: 9.4 ± 7.1 mg/dL, *P* < 0.001), TG (delta change: −35.8 ± 44.1 mg/dL *P* < 0,001), HbA_1__c_: (delta change: −1.2 ± 1.6% [−10 ± 6 mmol/mol], *P* < 0.001), HOMA-IR (delta change: −4.7 ± 3.9 mg/dL, *P* < 0.001), and CRP (delta change: −8.5 ± 6.7 μg/mL *P* < 0.001).

**FIGURE 1:**
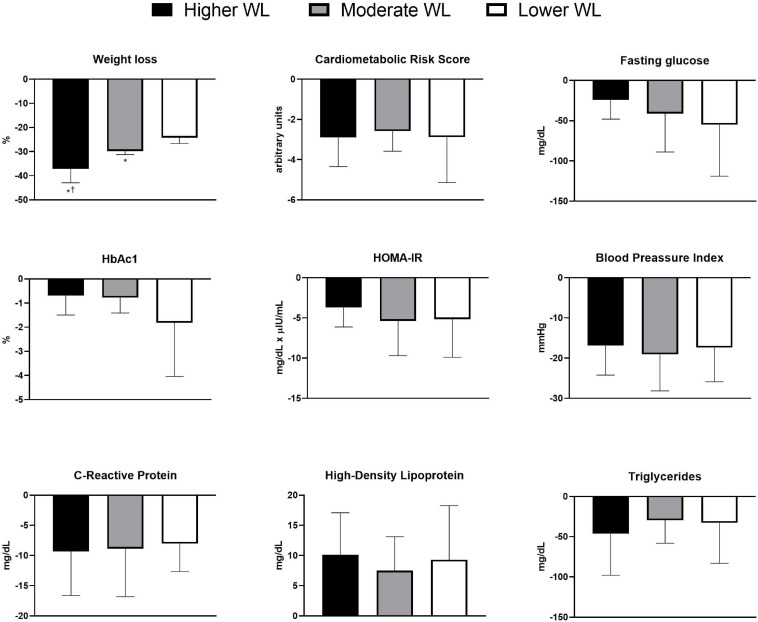
Weight loss, cardiometabolic risk score, fasting glucose, glycated hemoglobin, homeostasis model assessment, blood pressure index, C-reactive protein, high-density lipoprotein, and triglycerides 9 months following surgery in higher, moderate, and lower weight loss groups. WL = weight loss. *P < 0.05 vs. lower weight loss; ^†^*P* < 0.05 vs. moderate weight loss.

**FIGURE 2:**
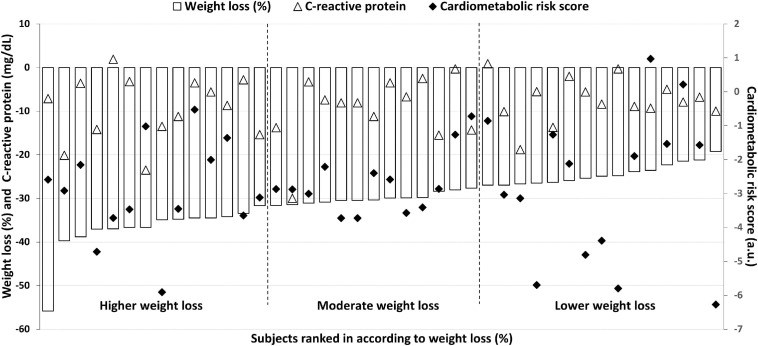
Individual data for changes in body weight, clustered cardiometabolic risk score and C-reactive protein 9 months following surgery. Benefits in the cardiometabolic risk and C-reactive protein is observed across the spectrum of changes in body weight, indicating that magnitude of weight loss is not determinant for improvements in cardiometabolic risk factors.

There were no differences in cardiometabolic risk score, fasting glucose, HbA_1__c_, HOMA-IR, blood pressure index, CRP, HDL, and TG between tertiles (Figure 1). Figure 2 illustrates individual data for changes in body weight, CRP and clustered cardiometabolic risk. Individual inspection confirms cardiometabolic improvements across the weight-loss spectrum. Weight loss was negatively associated with HbA1c (*r* = −0.351, *P* = 0.023). No other significant associations were found (all *P* > 0.05) (Table 2).

**TABLE 2:** Association between weight loss 9 months following surgery and cardiometabolic parameters.

	**Weight loss (%)**	
	***r***	***P***
Clustered cardiometabolic risk	0.049	0.759
Fasting glucose	–0.274	0.079
HbA_1__c_	–0.351	0.023
HOMA-IR	–0.203	0.202
Blood pressure index	–0.002	0.987
C-reactive protein	0.096	0.544
HDL	–0.141	0.374
Triglycerides	0.082	0.608

*HbA_1c_: glycated hemoglobin; HOMA-IR: homeostasis model assessment; HDL: high-density lipoprotein; LDL: low-density lipoprotein; TG: triglycerides.*

Similar results were found in the sensitivity analysis considering changes in weight and cardiometabolic parameters from PRE to POST3. There were no differences in any cardiometabolic parameters between the tertiles (Supplementary Figure 2), nor were there significant correlations between changes in weight and changes in cardiometabolic outcomes (Supplementary Table 2).

## Discussion and Conclusion

Our findings demonstrate that the percentage total weight loss did not promote any differences in cardiometabolic risk score, fasting glucose, TG, HbA1c, HOMA-IR, CRP, and blood pressure index. This suggests that health-related improvements brought about by bariatric surgery go beyond the magnitude of weight loss *per se*, casting doubt on the use of this parameter alone to determine the clinical success of the surgical procedure.

There is no doubt that excess of body weight is associated with comorbidities and poor health outcomes, but exclusive focus on body weight in the management of obesity has been increasingly criticized (Ross et al., 2000, 2020). Weight loss has also been used as a marker of success for patients with morbid obesity underdoing bariatric surgery (Maggard et al., 2005). Herein, we tested whether cardiometabolic improvements following surgical treatment would be possible across different weight-loss responses. Our findings indicate that this provides relevant and important data for several key outcomes. These findings support the view that body weight alone does not precisely reflect the effectiveness of weight-management interventions (Ross et al., 2015; Hyde et al., 2019), extending this conclusion to surgical treatments. Thus, our data align with the proposition that health care practitioners managing obesity should target broader cardiometabolic risk factors rather than body weight alone (Ross and Janiszewski, 2008; Ross et al., 2015). In this regard, it was recently demonstrated that patients after obesity remission following gastric bypass with higher body fat percentage had lower insulin sensitivity and higher triglyceride levels, independent of their BMI. It was concluded that assessing body fatness (as assessed by DXA) provides helpful information on metabolic health in non-obese patients after gastric bypass (Eriksson Hogling et al., 2020). This is partially supported by *post hoc* analysis (Pearson product–moment linear correlation) in which we found a significant association between fat mass loss (as assessed by DXA, as described in Murai et al., 2019) and changes in clustered cardiometabolic risk factors (*r* = 0.328, *P* = 0.044).

This study has limitations. First, this was an ancillary analysis derived from a two-arm trial involving individuals engaged or not to exercise. As this was an exploratory study with a limited sample size, we did not run sub-analysis for each group to determine the extent that the exercise intervention may contribute to the findings. However, as groups were comparable for the outcomes investigated, we do not think that exercise training was a factor. Second, the follow-up was relatively short, which limits conclusions about the longer-term relationships between weight loss groups on cardiometabolic risk factor. This is important because patients may experience weight rebound in the long-term, which may affect cardiometabolic risk factors in a way that would be quite different to that observed in the current study. Third, only women were selected since they correspond to roughly 90% of the patients followed by our Bariatric Surgery Unit. We felt that including a few men in the study would increase sample heterogeneity and, hence, add complexity to interpretations and conclusions. Finally, the minimum weight loss observed within our sample was around 20%, which is higher than that reported in previous behavioral or pharmacotherapy trials (Alger et al., 1999; Fogelholm et al., 1999; Norris et al., 2004; Seimon et al., 2019). Importantly, these interventions have been associated with cardiometabolic improvements, despite the smaller weight loss (Alger et al., 1999; Fogelholm et al., 1999; Seimon et al., 2019). This might suggest that the magnitude of weight loss seen in this study may have exceeded the minimum weight loss needed to elicit cardiometabolic improvements.

In conclusion, the percentage weight loss promoted by bariatric surgery is not *per se* related to improvements in cardiovascular risk factors. If these findings are confirmed by larger trials, the use of weight loss alone may be deemed inadequate to determine surgery success. The identification of new biomarkers that could better predict surgery success remains as an unmet need.

## Data Availability Statement

The raw data supporting the conclusions of this article will be made available by the authors, without undue reservation.

## Ethics Statement

The studies involving human participants were reviewed and approved by the Ethics Committee of Clinical Hospital of the School of Medicine of the University of São Paulo. The patients/participants provided their written informed consent to participate in this study.

## Author Contributions

HR and BG had full access to all data in the study, took responsibility for the integrity of the data and the accuracy of the data analysis, and supervised the study. BG, HR, and SG designed the research. All authors conducted the research and involved in critical revision of the manuscript for important intellectual content. BG, HR, SG, and KG drafted the manuscript. SG and KG did statistical analysis.

## Conflict of Interest

The authors declare that the research was conducted in the absence of any commercial or financial relationships that could be construed as a potential conflict of interest.
