# Supplementary material for: Constraints of Weight Loss as a Marker of Bariatric Surgery Success: An Exploratory Study
Source: Front Physiol. 2021 Jun 11;12:640191. doi: 10.3389/fphys.2021.640191 (PMC8232885; doi:10.3389/fphys.2021.640191)

**SUPPLEMENTARY TABLE**

|  | **Table S1.** Comparisons between delta changes (9 months after surgery - baseline) in non-exercise (RYGB) and exercise (RYGB+ET) groups. | | | | |
| --- | --- | --- | --- | --- | --- |
|  |  | **RYGB**  **(n = 21)** | | **RYGB + ET**  **(n = 21)** | **Between-group**  **difference (P value)** |
|  | Weight loss, % | | -30.4 ± 5.3 | -30.3 ± 7.6 | -0.1 (0.9663) |
|  | Clustered cardiometabolic risk, a.u. | | -2.5 ± 1.7 | -3.0 ± 1.5 | 0.5 (0.3417) |
|  | Fasting glucose, mg/dL | | -47.8 ± 56.4 | -32.6 ± 39.1 | -15.2 (0.3167) |
|  | HbA_1c_, % | | -1.2 ± 1.9 | -1.0 ± 1.0 | -0.2 (0.6576) |
|  | HOMA-IR | | -4.3 ± 3.3 | -5.1 ± 4.4 | -0.7 (0.5652) |
|  | Blood Pressure index, a.u. | | -16.1 ± 9.2 | -19.6 ± 6.8 | 3.4 (0.1850) |
|  | C-Reactive Protein, mg/dL | | -7.0 ± 5.4 | -10.5 ± 7.4 | 3.5 (0.0882) |
|  | HDL, mg/dL | | 7.4 ± 7.7 | 10.6 ± 6.4 | -3.2 (0.1541) |
|  | Triglycerides, mg/dL | | -32.1 ± 38.9 | -40.3 ± 49.6 | 8.2 (0.5546) |
| **Data are expressed as mean ± SD. HbA_1c_: glycated hemoglobin; HDL: High-Density Lipoprotein; LDL: Low-Density Lipoprotein; a.u.: arbitrary unit.** | | | | | |

| **Table S2.** Association between weight loss 3 months following surgery and cardiometabolic parameters. | | |
| --- | --- | --- |
|  | **Weight loss (%)** | |
|  | ***r*** | ***P*** |
| Clustered cardiometabolic risk | 0.068 | 0.669 |
| Fasting glucose | 0.254 | 0.104 |
| HbA_1c_ | 0.187 | 0.236 |
| HOMA-IR | -0.192 | 0.458 |
| Blood Pressure index | -0.033 | 0.834 |
| C-Reactive Protein | -0.031 | 0.848 |
| HDL | 0.215 | 0.171 |
| Triglycerides | 0.061 | 0.700 |
| HbA_1c_: glycated hemoglobin; HDL: High-Density Lipoprotein; LDL: TG: Triglycerides. | | |

**SUPPLEMENTARY FIGURE**

Figure S1. CONSORT flow diagram of the study.

**Figure S2.** Weight loss, cardiometabolic risk score, fasting glucose, glycated hemoglobin, homeostasis model assessment, blood pressure index, C-reactive protein, high-density lipoprotein, and triglycerides 3 months following surgery in higher, moderate, and lower weight loss groups. WL = weight loss. * indicates *P*<0.05 vs. lower weight loss; † indicates *P*<0,05 vs. moderate weight loss.

**Allocation**

**Analysis**

**Follow-Up**

**Enrollment**

Assessed for eligibility (n = 222)

Excluded (n = 168)

♦  Not meeting inclusion criteria (n = 100)

♦  Declined to participate (n = 60)

Evaluated by complete case analysis (n = 21)

Lost to follow-up (n = 10)

♦ Did not undergo surgery (n = 3)

♦ Withdrew after surgery for personal reasons (n = 7)

Allocated to RYGB (n = 31)

Lost to follow-up (n = 9)

♦ Did not undergo surgery (n = 4)

♦ Withdrew after surgery for personal reasons (n = 5)

Missing data for cardiometabolic outcomes (n = 1)

mii

miss

Allocated to RYGB+ET (n = 31)

Evaluated by complete case analysis (n = 21)

Randomized (n = 62)


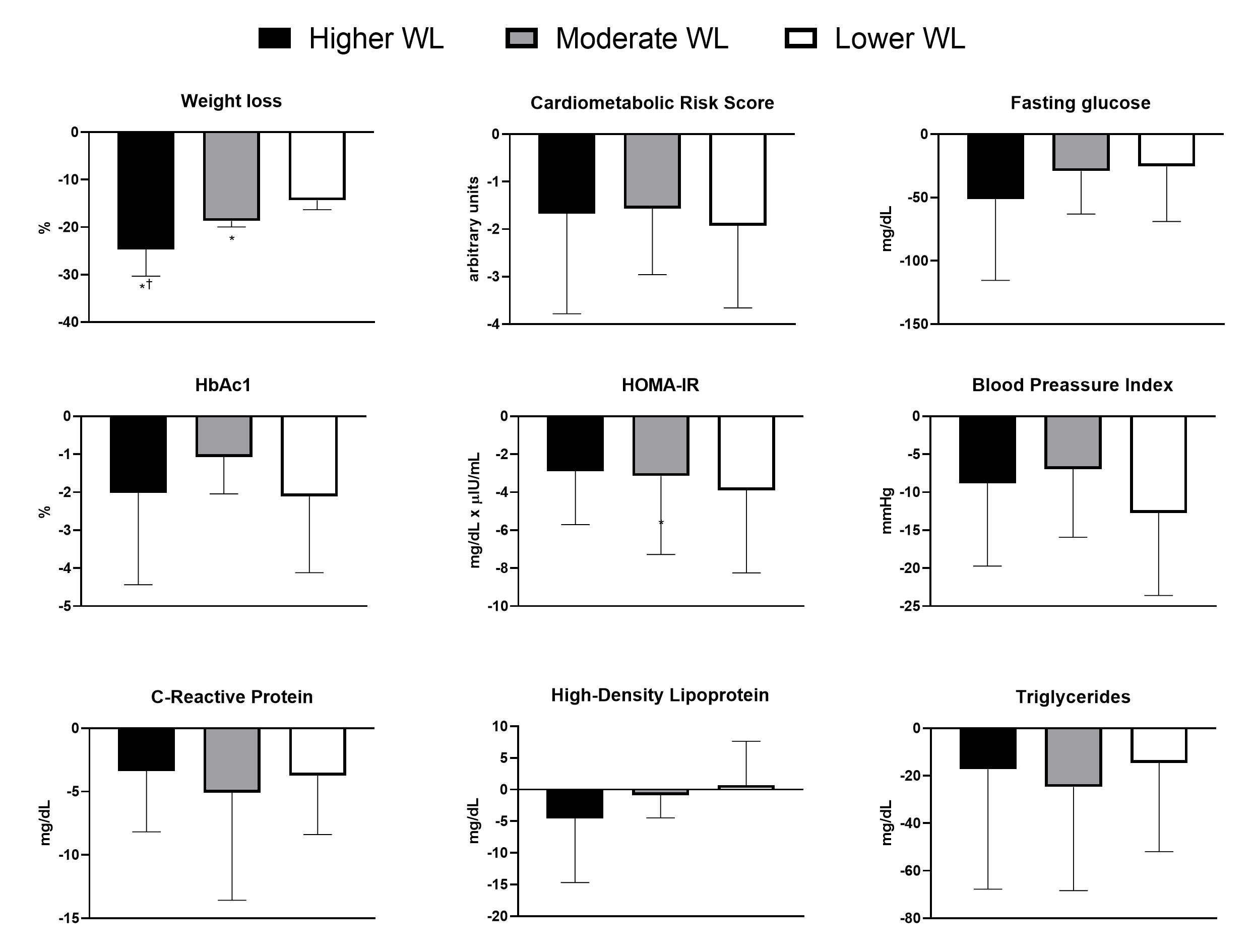

Supplement: Supplementary file 1 [file Data_Sheet_1.doc]
